# The combined effects of treated sewage discharge and land use on rivers

**DOI:** 10.1111/gcb.16934

**Published:** 2023-09-21

**Authors:** Dania Albini, Lauren Lester, Philip Sanders, Jocelyne Hughes, Michelle C. Jackson

**Affiliations:** ^1^ Department of Biology University of Oxford Oxford UK; ^2^ Somerville College University of Oxford Oxford UK; ^3^ School of Geography and the Environment University of Oxford Oxford UK

**Keywords:** agriculture, algae, freshwater, macroinvertebrates, multiple stressors, nutrients, treated sewage, urbanisation, water quality

## Abstract

Freshwater ecosystems are increasingly threatened by multiple anthropogenic stressors. Release of treated sewage effluent and pollution from agricultural or urban sources can independently reduce water quality with implications for ecological communities. However, our knowledge of the combined effects of these stressors is limited. We performed a field study to quantify the combined effect of treated sewage discharge and land use on nutrient concentrations, sewage fungus presence and communities of macroinvertebrates and benthic algae. Over three seasons in four rivers we found that a model which included an interaction between sewage pollution and time of the year (i.e. months) was the best predictor of nutrient concentrations and the abundance of algae and sewage fungus. Both macroinvertebrate and algae communities shifted downstream of sewage input. Specifically, more tolerant groups, such as cyanobacteria and oligochaetes, were more abundant. The EPT (Ephemeroptera, Plecoptera and Tricoptera) water quality score was best explained by an interaction between month and agriculture in the surrounding landscape. Overall, our results show that sewage discharge has a significant impact on water quality and benthic riverine communities, regardless of the surrounding land uses. Agricultural inputs, however, could be more important than treated sewage discharge in reducing the abundance of sensitive invertebrate taxa. We need both improvements to wastewater treatment processes and reductions in agricultural pollution to reduce threats to vulnerable freshwater communities.

## Graphical abstract

Freshwater ecosystems face great threats from multiple stressors. Sewage effluent and agricultural/urban land uses can independently degrade water quality, impacting aquatic ecosystems. Yet, combined effects remain poorly understood. Our study evaluated the effects of sewage and land use on nutrients, sewage fungus and riverine biodiversity. Across four rivers and three seasons, the interplay between sewage and time emerged as the best predictor for nutrient levels, algae and sewage fungus. Downstream regions exhibited greater abundance of resilient macroinvertebrates and algae. The EPT score reflected the interplay between month and agricultural land use. Treated sewage affected water quality and benthic life, and agriculture affected sensitive macroinvertebrate species.

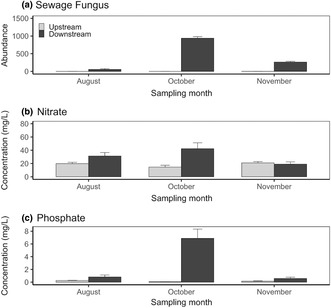

## 1 INTRODUCTION

In the last few decades, pollution of aquatic ecosystems has become an environmental issue of global concern (Dulio et al., 2018; Luo et al., 2014; Schaar et al., 2010). In particular, wastewater inputs have been recognised as one of the most common threats to water quality in river ecosystems (Turner et al., 2003; Whelan et al., 2022). Despite treatments in wastewater plants (WWPs) to remove some suspended solids and nutrients, these are not entirely removed and other chemical pollutants (e.g. pharmaceuticals) still enter aquatic ecosystems (Luo et al., 2014; Whelan et al., 2022). These substances can drastically affect the rivers physical and chemical characteristics (e.g. low pH and high nutrients, e.g. Curtis & Harrington, 1971; Geatches et al., 2014; Oenemaa et al., 2005), with consequences for the survival and abundance of aquatic organisms (e.g. macroinvertebrates and algae communities, Chambers & Prepas, 1996; Guzman et al., 2023; Wright et al., 1995). The discharge of effluent into rivers has also been related to the development of ‘sewage fungus’, a complex matrix of fungus filaments, algae and bacteria (Mason, 1996) that can interact in unpredictable ways with the riverine community.

Pollution from agricultural and urban inputs is rising due to the fast increase in global population and per capita resource consumption (Feng et al., 2023; Mohiuddin et al., 2011; Zhang et al., 2023), but the combined effect of pollution from both treated sewage input and land use on river communities is largely unexplored (Cooper, 1993; Mohiuddin et al., 2011; Neumann & Dudgeon, 2002). Runoff from agricultural fields introduces sediment, organic matter and fertilisers into streams and rivers, changing their water quality in terms of nutrients, primarily phosphorus (P) and nitrogen (N) (Cooper, 1993; Neumann & Dudgeon, 2002). Both treated sewage input and agricultural land use increase concentrations of bioavailable phosphorus forms. Freshwater ecosystems are usually phosphorus limited, so this can cause elevated algae growth rates leading to eutrophication (Mainstone & Parr, 2002; Slavik et al., 2004). Contamination from urban areas is also rapidly increasing due to, for example, road runoff entraining chemicals and particulates when heavy rain occurs (Mohiuddin et al., 2011; Wildi et al., 2004). Rivers are thus often subjected to a variety of different stressors that can interact with each other. However, studies that explore the interaction from multiple pollutant sources (i.e. from treated sewage discharge, agriculture and urban runoff) are rare. There is an urgent need to consider diffuse sources of pollution (i.e. runoff from land use) to better understand the relative contribution of wastewater effluents as point sources of pollution. This will promote better management decisions, regulation and legislation (Ding et al., 2015; Jarvie & Neal, 2006; Stutter & Cains, 2017).

The most widely used biomonitoring approaches for river water quality are based on changes in benthic macroinvertebrate community structure and abundance, for example, macroinvertebrates have been used to assess effluent contamination (Growns et al., 1995; Wright et al., 1995), and urban and agricultural land use (Chessman & Williams, 1999; Gresen et al., 2007; Neumann & Dudgeon, 2002; Walsh et al., 2001). Macroinvertebrates are a group of organisms that play a key role in river ecosystems and show large variation in their sensitivities to anthropogenic stressors (Sumudumali & Jayawardana, 2021; Wallace & Webster, 1996). Modification of the benthic algae community (i.e. periphyton), which are at the base of the food web, could also be used as an indicator of water quality (Rosenberger et al., 2008), because as algal communities change, the whole food web can also restructure (e.g. Hampton et al., 2011; Rosenberger et al., 2008).

Here, we investigate the single and interactive effects of three different pollution sources, namely treated sewage discharge, agricultural land use and urban land use in four English rivers, over three seasons. Analyses of two main nutrient changes (i.e. nitrate and phosphate), algal and benthic invertebrate responses, alongside quantification of sewage fungus were combined with land use data, to gain a comprehensive overview of pollutant effects on river water quality. We hypothesised that the effects of treated sewage discharge would dominate over the effects of background land use pollution.

## 2 METHODS

This research was performed during three sampling campaigns (August, October and November), at four rivers in England (the United Kingdom) in 2021 (Figure S1). All rivers were sampled in two areas: (1) 5–10 m upstream (controls) and (2) 5–10 m downstream (impacts) of the point of a treatment sewage discharge input. Sampling distance was chosen to ensure the detection of the effects of treated sewage discharge, both upstream and downstream (if any) and following the literature (Giebułtowicz et al., 2016; Grover et al., 2011). Anonymity of the river locations has been intentional to protect the identity of the wastewater treatment company who permitted us access to their treatment works.

### 2.1 Nutrients and sewage fungus

Three replicates of 100 mL of water were randomly collected from each sample area (i.e. upstream and downstream of the treated sewage input) at each sampling occasion to analyse nutrients including nitrate ($NO_{3}^{-}$), phosphate ($PO_{4}^{3-}$), chloride (${\mathrm{Cl}}^{-}$) and sulphate ($SO_{4}^{2-}$) concentrations. Water samples were kept refrigerated and analyses were undertaken in the laboratory of the School of Geography and the Environment at the University of Oxford (methods in Table S1; Figure S1). To estimate sewage fungus abundance (Figure S1c, Albini et al., 2023), two further 50 mL water samples were preserved using three drops of 25% glutaraldehyde (see Table S1 for nutrients and Methods in Supplementary material for estimation of sewage fungus abundance).

### 2.2 Algae and macroinvertebrates

Biomass of benthic algae was quantified in triplicates on the first three sampling occasions using rocks collected at the bottom of the streams, with a BenthoTorch (bbe Moldaenke, Germany). The BenthoTorch enables real‐time quantification of benthic algal concentrations (green algae, cyanobacteria and diatoms) by measuring chlorophyll‐a fluorescence over a surface of 0.78 cm^2^ (Rosero‐López et al., 2021).

To collect macroinvertebrates, we took three Surber samples (25 × 25 cm area, 250 μm mesh size, Figure S1) from each sample area at each sampling occasion. These were preserved in 95% ethanol before sorting, identifying and counting in the laboratory using the Riverfly identification key for the United Kingdom (*Extended Riverfly 33* Scheme, http://www.riverflies.org). The Extended Riverfly methodology is a citizen science tool used to classify riverine macroinvertebrates into broad groups based on their taxonomy as well as responses to stressors (nutrient enrichment, flow rates, sedimentation and acidification). This method is comparable to abundance data obtained with conventional research practice (Krabbenhoft & Kashian, 2020). We also calculated the Ephemeroptera, Plecoptera and Tricoptera (EPT) score, which indicates the abundance of three sensitive groups: Ephemeroptera (mayflies), Plecoptera (stoneflies) and Trichoptera (caddisflies) (Lenat, 1988).

### 2.3 Land use classification and analysis

Google Earth Engine (GEE) was used to complete the land cover classification. Through the open‐source interface, the *Sentinel‐2* satellite data for the sampling period were scoured and used as input. A rectangular region of ∼ 2150 km^2^ was created to include the upstream area of each impact site, with the outflow point of the treated sewage being at the bottom of the region. The distance from the outflow point to the top side of the rectangular region was approximatively of 940 m in length. This area was chosen to reflect the local upstream catchment of each site. To generate land cover classifications in GEE, a supervised classification was completed over the ‘Feature Collection’ (i.e. the imagery from the *Sentinel‐2* satellite). To train the classifier, sections of known features were used to create libraries (Gorelick et al., 2017), producing an overlay map that delineated the new land cover classifications using normalised difference vegetation index (NDVI) bands (Digavinti & Manikiam, 2021). For this study, two land cover types were defined: ‘agriculture’ and ‘urbanisation’ (Table S2). The agricultural category represented areas with fallow, irrigation, grazing land and active crops, while the urban category included large industrial regions and residential suburbs. Percentages of the two land cover types were then derived for each polygon.

### 2.4 Statistical analysis

Statistical analyses were performed using R‐studio (version 1.4.17, Rstudio Team, 2020). We used linear models to test for an effect of presence of treated sewage discharge (i.e. area of sampling: upstream or downstream) and land use (i.e. urbanisation and agriculture) on the river community (periphyton and macroinvertebrate abundance and indices), abundance of sewage fungus and nutrient concentrations (using the *lme4* package, Bates et al., 2015). Month, per cent urban and agricultural land use and the presence of treated sewage discharge were our fixed effects, and sites were included as a random effect, producing a random intercept for the model. We ran models for individual, additive and interactive effects of all single drivers and driver pairs and selected the best model based on Akaike information criterion (AIC). The significance of the models was tested by performing a comparison with a null model (i.e. a model where we removed all fixed effects except for the intercept), and conditional *R* ^2^ (which included the effect of the random term) values were calculated for the best models using the *r.squaredGLMM* package (Nakagawa & Schielzeth, 2013). Detrended correspondence analysis (DCA) in the *vegan* package (Oksanen et al., 2019) was used to explore variation in benthic community structure. We used square root transformed relative abundance data to dampen the influence of outliers and the 10% most important species in driving the trend were plotted using the *ordiselect* function. The most important environmental variables were passively overlaid.

## 3 RESULTS

### 3.1 Nutrients and sewage fungus

**FIGURE 1:**
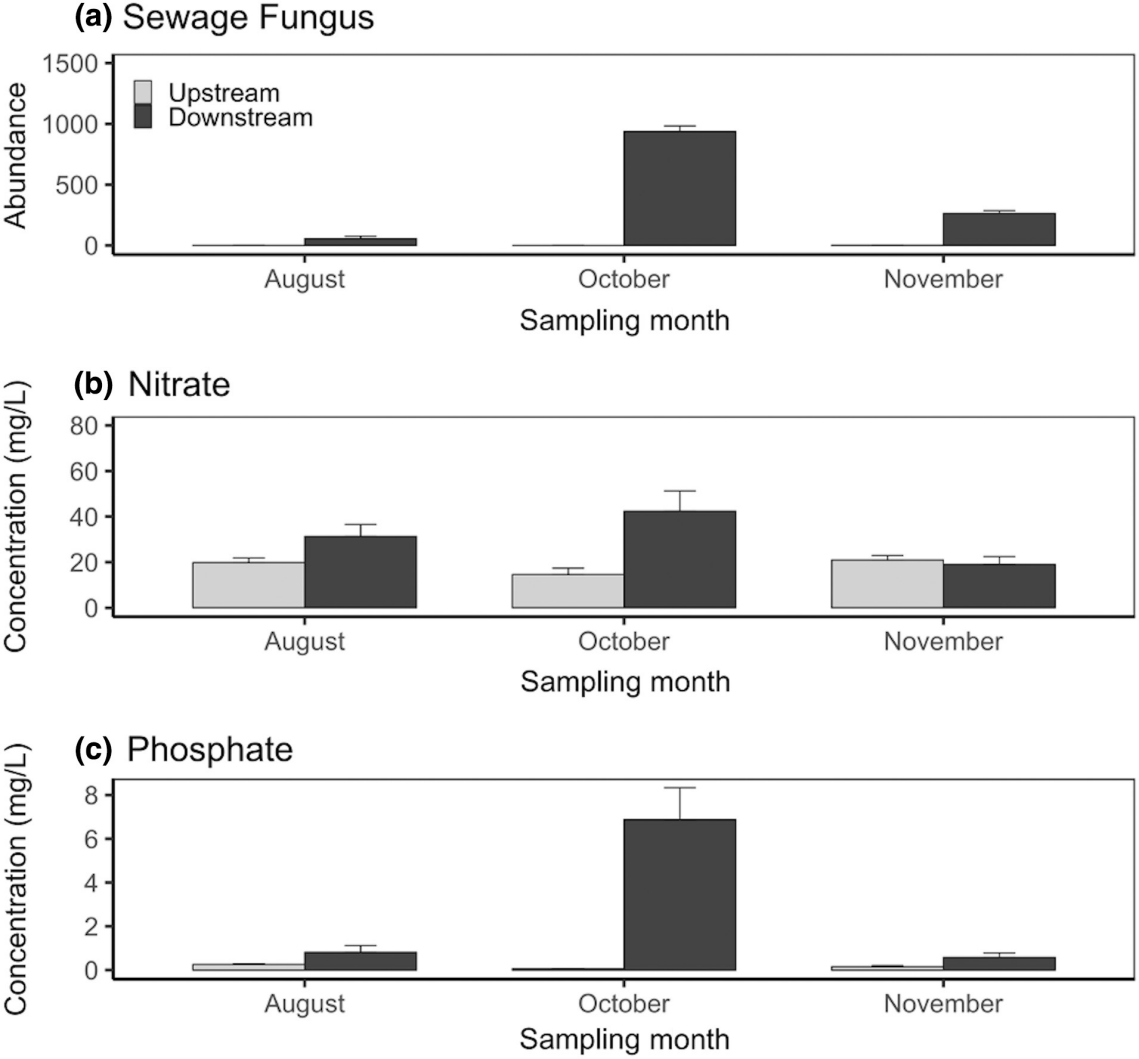
Sewage fungus abundance (filaments/20 mL), (a) and nutrients concentration (mg/L), (b) and (c) in the river areas with (downstream, in dark grey) and without (upstream, in light grey) treated sewage discharge over the sampling months. Bars indicate the standard errors.

The best model to predict nutrient concentration and sewage fungus abundance included an interaction between sampling month and treated sewage discharge (Table S3). We detected a higher concentration of nitrates, phosphate and sewage fungus in the downstream river areas which received treated sewage input and in the sampling month of October (Figure 1).

### 3.2 Algae and macroinvertebrates

**FIGURE 2:**
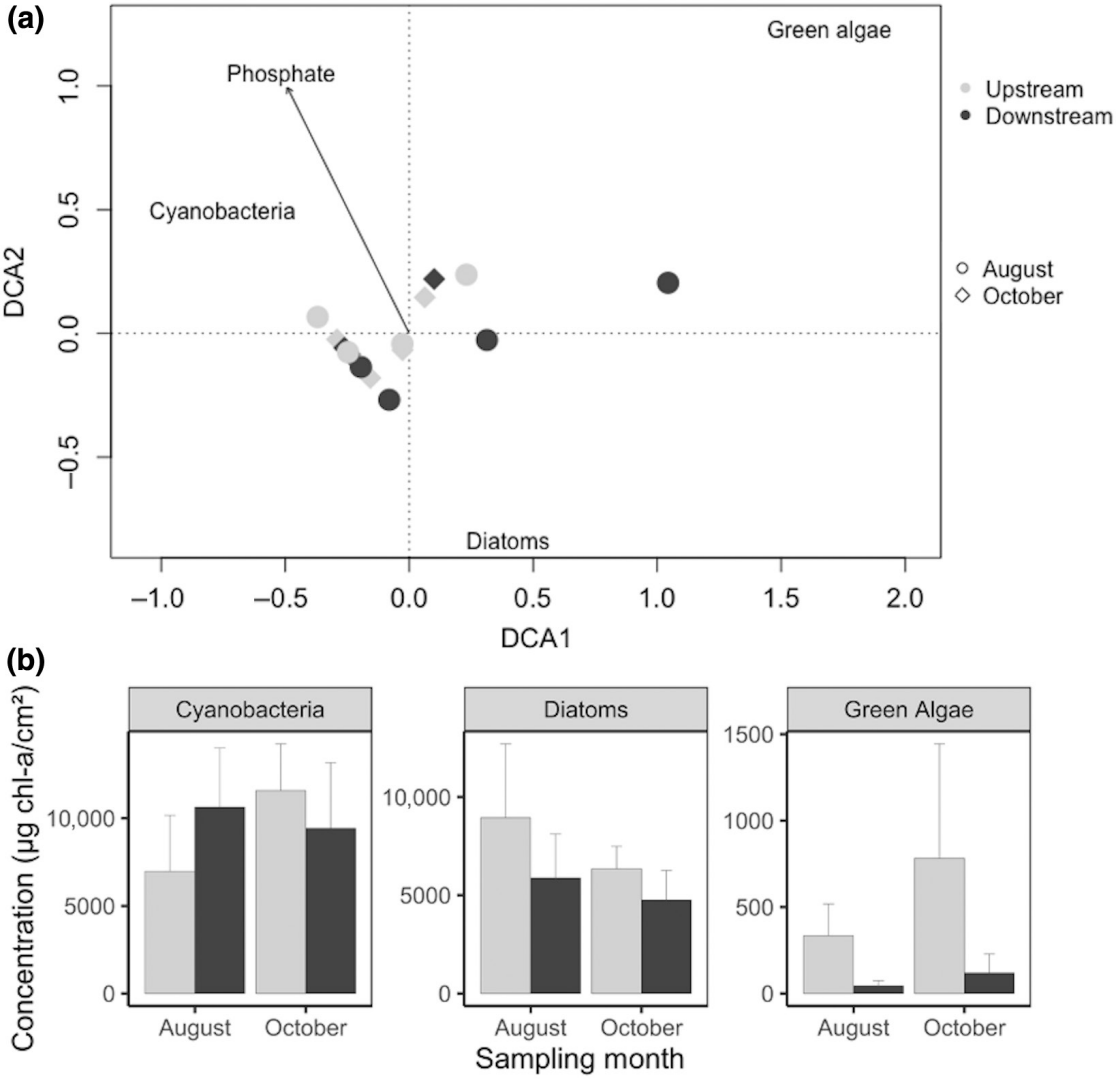
(a) Detrended correspondence analysis plot for benthic algae species (i.e. diatoms, green algae and cyanobacteria), where each point represents the community in a single river area. Symbols represent the sampling months and colours represent the absence (upstream—light grey) and presence (downstream—dark grey) of treated sewage discharge. Environmental variables are passively overlaid on the plot: no variables were significant and therefore only the most important variable—phosphorus—is included. (b) Mean abundance ± SE of benthic algae, in the areas without (upstream) and with (downstream) treated sewage input, over the sampling month.

Variation in the abundance of total benthic algae was associated with an interaction of presence of treated sewage discharge and month of sampling (Table S3). Detrended correspondence analysis (DCA) revealed separation between the communities exposed to treated sewage discharge (i.e. downstream, Figure 2a) and those upstream. Green algae and diatoms abundance were higher in the river area without treated sewage discharge, while cyanobacteria exhibited greater tolerance to the sewage discharge, and they dominated the downstream area in terms of overall abundance (Tables S3 and S4; Figure 2b).

**FIGURE 3:**
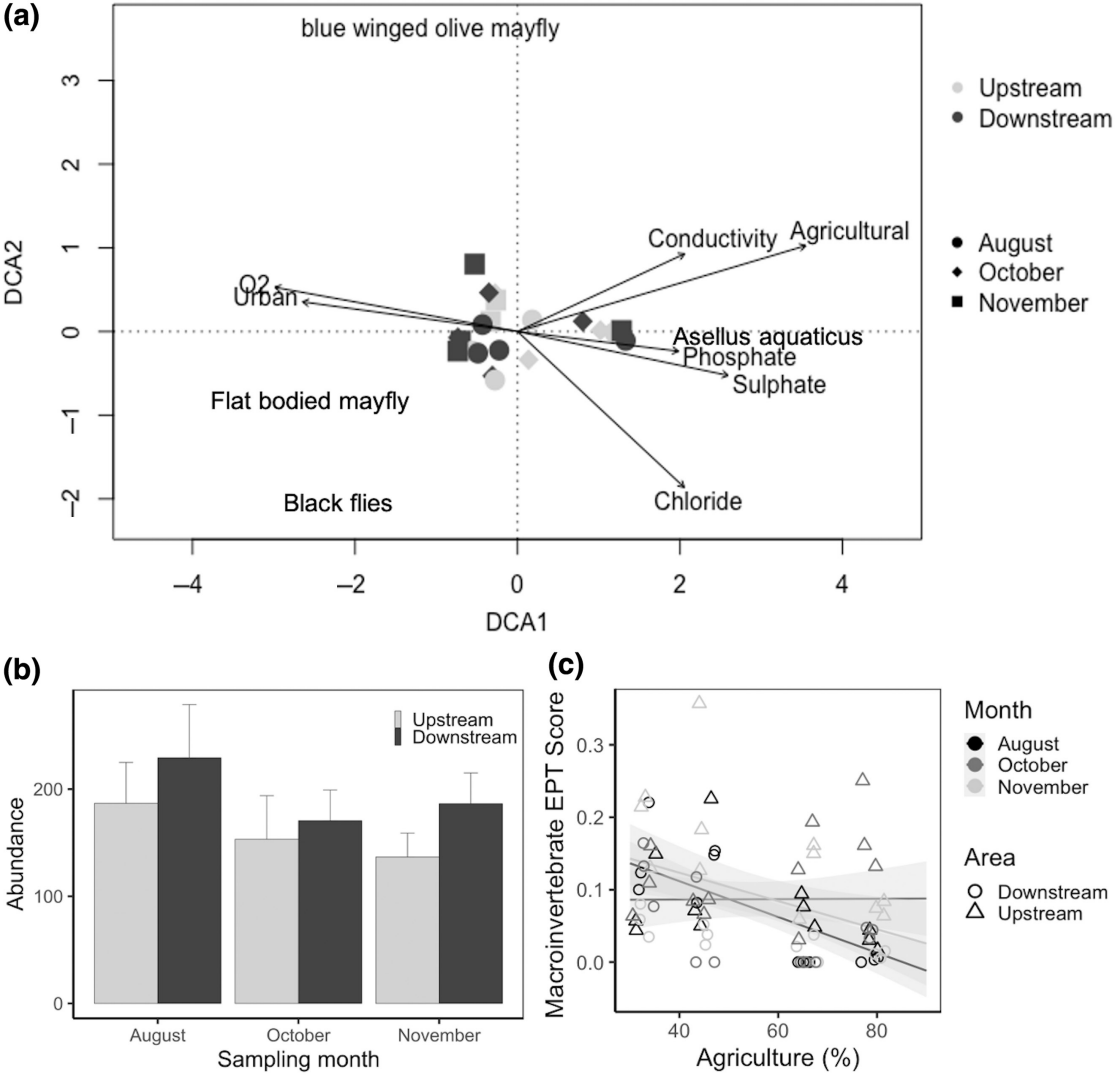
(a) Detrended correspondence analysis plot for macroinvertebrate groups, where each point represents the community in a single river area. Symbols represent the three sampling months and colours represent the absence (upstream—light grey) and presence (downstream—dark grey) of treated sewage discharge. The 10% most important species driving the trends are shown, and significant (*p* < .05) environmental variables are passively overlaid on the plot. (b) Mean abundance ± SE of macroinvertebrates, in the areas without (upstream) and with (downstream) treated sewage discharge, over the sampling months. Abundance is expressed as individuals per 500 mL. (c) Decrease in the macroinvertebrate EPT score with an increase in the percentage area of agriculture. Colour of the symbol represents the month of sampling (black = August, dark grey = October, light grey = November) and shapes differ with the absence (triangles = upstream) or presence (circles = downstream) of treated sewage discharge.

Average abundance of all macroinvertebrates was higher downstream of treated sewage input (Figure 3b), and dominated by *Gammarus* spp., oligochaetes and cased caddisfly (accounting for 38%, 14% and 12% respectively). *Gammarus* spp. and coleoptera (adults and larval stages) were the most abundant groups (34% and 12% respectively) upstream. Abundance was best explained by an interaction between treated sewage discharge and month (Table S3) and there was a separation between the communities in the presence or absence of treated sewage discharge (Figure 3a). There was an interactive effect of agriculture and sampling month on the EPT score, with the former causing an overall score decline, which was steeper in August (Figure 3c; Table S3). Results of the second macroinvertebrate index, the Riverfly score, was best explained by the model with the random intercept only (Table S3).

## 4 DISCUSSION

Our results show that treated sewage discharge played a greater role than land use in driving changes in water quality and benthic communities, supporting our initial hypothesis. Among our response variables, only EPT score was best predicted by agricultural land use. This suggests that water quality and benthic organisms are generally more threatened by treated sewage discharge than pollution from the surrounding catchment.

We found an increase in nutrients and sewage fungus downstream of treated sewage input which caused higher cyanobacteria abundance, while green algae and diatoms declined. The time of sampling (i.e. months) was also significant, with the October peak in nutrient concentrations is most likely explained by a storm overflow event (i.e. the legal release of untreated sewage in rivers when high rain occurs) which happened a week before we sampled. This caused higher cyanobacteria concentrations in October, while diatoms exhibited an opposite trend. Specifically, diatom densities increased in August while cyanobacteria were still in high concentration but less dominant. Globally, cyanobacteria are well known for their ability to produce secondary toxic metabolites, which are responsible for the death of many aquatic organisms (e.g. fish and zooplankton, Ernst et al., 2006), with the potential to significantly alter the structure of native aquatic communities (Pietsch et al., 2001; Salmaso et al., 2015; Sukenik et al., 2015). Cyanobacteria have been previously found to metabolise and degrade heavy metals and xenobiotics (Kumar & Sahu, 2012) and to proliferate in environments with high phosphorous levels such as treated sewage effluent (Kruk et al., 2023; Vasconcelos & Pereira, 2001; Figure 3a). The presence of high cyanobacteria densities in rivers can lead to the displacement of eukaryotic algae (Salmaso et al., 2015). Hence, the decrease in diatoms downstream of the treatment plant could potentially be attributed to the presence of cyanobacteria. Alternatively, the turbidity produced by the discharge causing shading or an increase in growth of competing organisms might be responsible for diatom declines (Masseret et al., 1998; Salmaso et al., 2015; Welch et al., 1992). The input of ammonia by a sewage effluent can also inhibit the growth of some species of diatoms and green algae, either by producing changes in the chloroplasts (Kaown et al., 2023; Kohl & Nicklisch, 1988), or by a toxic effect inhibiting photophosphorylations (Hürlimann & Schanz, 1993).

Total macroinvertebrate abundance was higher in the river areas with treated sewage discharge for all sampling months, with higher abundance in August. Previous studies have shown a similar pattern, due to a combination of increased nutrient concentrations in the discharge and higher temperatures in summer months, which favour tolerant macroinvertebrate proliferation (Hamdhani et al., 2020; Jesus et al., 2020; Rodríguez‐Castillo et al., 2017). Kownacki and Szarek‐Gwiazda (2022) found that in the heavily polluted lowland of the sewage‐contaminated Vistula River (Poland), macroinvertebrate density was high due to the dominance of Oligochaeta, a group which is highly tolerant of organic toxicants (see also Hamdhani et al., 2020). Our study supports this trend—we found that the elevated abundance of macroinvertebrate in the downstream area was caused mainly by the tolerant groups of oligochaetes, *Gammarus* spp., craneflies, non‐biting midge and water beetles. In contrast, sensitive species such as stoneflies and the blue winged olive mayfly (*Baetis*), were more prevalent in the absence of treated sewage discharge (Everall et al., 2018; Johns & Benning, 2021). Loss of some macroinvertebrate species due to wastewater pollution could alter or degrade critical ecosystem processes, such as the detrital decomposition of organic matter, because of the unavailability of replacement species (e.g. Burdon et al., 2020; Jackson et al., 2020; Wallace & Webster, 1996; Wilson, 1992, but see Benelli et al., 2018). While the EPT score was best explained by agriculture in the surrounding catchment, declining as it increased, the Riverfly index revealed no significant effects by land use, or any other variables considered. This discrepancy could be explained by the fact that the first index focuses on sensitive taxa, while the second is an inclusive index of all riverine macroinvertebrates. Moreover, the EPT score had a sharper decline during the month of August, which corresponded to the period characterised by higher organism abundance. This association suggests that the density of macroinvertebrates may have influenced the decrease observed in the score. Using more than one water quality index and sampling in multiple seasons should, therefore, be encouraged when it comes to quantifying the effects of multiple stressors in rivers.

Changes in periphyton communities and macroinvertebrates due to wastewater contamination, could have potential significant environmental costs for higher trophic levels, with probable impacts on the overall flow of energy through the food web (Covich et al., 1999). A recent study (Carles et al., 2022) showed how periphyton communities exposed to wastewater shifted towards more sewage‐tolerant heterotrophic organisms. However, an increase in sewage‐tolerant periphyton species, or organisms in general, could result in enhanced sensitivity towards other stressors. This could cause changes in ecological functions, potentially leading to negative consequences for the ecosystem functioning and health. For instance, rates of leaf breakdown due to macroinvertebrate activities are a functional indicator of river health, and environmental stressors can negatively impact this function (Gessner & Chauvet, 2002; Young et al., 2008). Unexpected variation in breakdown rates might be caused by patchy distributions of particularly influential macroinvertebrate consumers due to anthropogenic stressors, such as sewage pollution. It is concerning that aquatic ecosystem functions may be irreversibly lost due to stressors, such as wastewater pollution: functional compensation may not occur by an increase in density of surviving species nor by an increase in processing rates of the few remaining tolerant species.

Aquatic ecosystems are frequently exposed to multiple stressors that interact with each other, leading to effects that are difficult to disentangle. With our study, by using a multi‐level novel approach which considers three of the most important threats to water quality in rivers, namely treated sewage discharge, and pollution from agricultural and urban areas, we have (a) highlighted the overarching extent to which river communities are negatively impacted by treated sewage discharge, and (b) demonstrated that agricultural pollution is the most important variable in reducing the abundance of sensitive macroinvertebrate taxa. Therefore, it is of paramount importance to understand the catchment processes that cause changes in river ecosystems because of land and water use. Furthermore, hydrological, geomorphological and ecological conditions of the rivers themselves could influence the response of river ecosystems to treated sewage discharge, which might not be uniform across different spatial sections. Using a spatially explicit approach, involving the strategic placement of sampling points at varying distances from the point of sewage discharge, can be an additional tool to provide valuable insights into the spatial extent and severity of the impact, by capturing data along the longitudinal gradient of the river. In general, to increase our understanding of the links between diffuse pollution sources at the catchment‐scale (such as runoff from land use) and direct point sources (such as wastewater pollution), we encourage the development of further studies who adopt this multi‐level approach, aimed to formulate management, regulatory and, ultimately, policy strategies to prevent continued degradation of freshwater communities in the United Kingdom.

## CONFLICT OF INTEREST STATEMENT

The authors declare no conflict of interest.

## Supporting information


Data S1

## Data Availability

The data that support the findings of this study are available as a supplementary material and on Dryad at https://doi.org/10.5061/dryad.00000008b.
