# Supplementary material for: The combined effects of treated sewage discharge and land use on rivers
Source: Glob Chang Biol. 2023 Sep 21;29(22):6415–22. doi: 10.1111/gcb.16934 (PMC10946937; doi:10.1111/gcb.16934)
Supplement: Supplementary file 1 — Data S1 [file GCB-29-6415-s001.doc]

**Supplementary material, Albini et al. 2023**

**Supplementary Method: Sewage fungus enumeration**

For quantifying sewage fungus filaments, we analysed 20 ml subsamples with a Bench Top FlowCAM® 8000 machine (Fluid Imaging Technologies, Inc., Maine, USA) with the particle analysis software (VisualSpreadsheet©, version 4). The FlowCAM® 8000 is an imaging flow cytometer that combines imaging and laser light to rapidly detect and count particles in a fluid sample. The FlowCAM settings included a 4× objective lens, 0.16 cm inner diameter tubing, a Field of View 300 flow cell (300 µm depth, 3000 µm width), an imaging rate of 21 frames/second, and a flow rate of 0.15 ml/min. AutoImage mode was utilized to process the captured images (Fluid Imaging Technologies Inc. 2011). Image libraries of sewage fungus were created by Albini D. prior to the experiment and used as a reference for identification with the FlowCAM. After photographing the samples, the VisualSpreadsheet© software automatically identified the images, which were manually verified for accuracy. The total number of images (i.e., filament density) was then recorded.

**Tables:**

**Table S1.** Methods used to analyse water chemistry and sewage fungus samples, at the Department of Geography (University of Oxford, UK)

| **Chemical Parameter** | **Preparation** | **Volume of sample** | **Technique** | **Make and model of kit** |
| --- | --- | --- | --- | --- |
| *Nitrate* | Filtered (0.45 µm) | 10 mL | Ion chromatography to determine anion concentration | ThermoScientific Dionex ICS-5000 chromatograph, with AS-AP autosampler |
| *Phosphate* |
|  |
| *Sewage fungus* | Filtered (250 µm) | 10 mL | Imaging rate of 21 frames/second; 119 and a flow rate of 0.15 ml/min. | FlowCam 8000 |

**Table S2.** Site Description

| Month | Site | Area | Urban | Agricultural | pH | O_2_  (mg/L) | Temperature (C) | TDS (mg/L) | Conductivity (µs/cm) | Sulphate (mg/L) | Nitrate (mg/L) | Phosphate (mg/L) |
| --- | --- | --- | --- | --- | --- | --- | --- | --- | --- | --- | --- | --- |
| August | impact1 | Downstream | 544.694378 | 960.73619 | 7.89 | 9 | 15.2 | 265 | 518 | 21.09 | 29.08 | 0.14 |
| August | impact1 | Downstream | 544.694378 | 960.73619 | 7.98 | 8.29 | 15.1 | 255 | 520 | 20.98 | 28.96 | 0.16 |
| August | impact1 | Downstream | 544.694378 | 960.73619 | 7.99 | 8.6 | 15.2 | 250 | 500 | 20.82 | 28.72 | 0.18 |
| August | impact1 | Upstream | 544.694378 | 960.73619 | 7 | 10.9 | 15.8 | 232 | 500 | 12.09 | 22.06 | 0.19 |
| August | impact1 | Upstream | 544.694378 | 960.73619 | 7.1 | 10.3 | 15.6 | 234 | 500 | 12.1 | 22.01 | 0.19 |
| August | impact1 | Upstream | 544.694378 | 960.73619 | 7 | 10.1 | 15.3 | 200 | 500 | 11.98 | 21.92 | 0.2 |
| November | impact1 | Upstream | 544.694378 | 960.73619 | 8.5 | 10.5 | 9.7 | 290 | 520 | 12.148 | 23.3351 | 0.045 |
| November | impact1 | Upstream | 544.694378 | 960.73619 | 8.4 | 10.3 | 10 | 281 | 500 | 12.2516 | 23.5058 | 0.0617 |
| November | impact1 | Upstream | 544.694378 | 960.73619 | 8.3 | 10.45 | 9.8 | 287 | 520 | 12.0706 | 23.3036 | 0.0699 |
| November | impact1 | Downstream | 544.694378 | 960.73619 | 7.82 | 9 | 10.2 | 312 | 500 | 20.9154 | 30.5528 | 0.1095 |
| November | impact1 | Downstream | 544.694378 | 960.73619 | 7.8 | 9.1 | 10.5 | 300 | 500 | 21.0141 | 30.7057 | 0.1106 |
| November | impact1 | Downstream | 544.694378 | 960.73619 | 7.81 | 9.2 | 9.1 | 301 | 500 | 21.1102 | 29.8563 | NA |
| October | impact1 | Upstream | 544.694378 | 960.73619 | 8.3 | 10.3 | 10 | 265 | 523 | 13.1478 | 21.1565 | 0.0456 |
| October | impact1 | Upstream | 544.694378 | 960.73619 | 8.2 | 10.3 | 11 | 270 | 520 | 13.053 | 21.5221 | 0.0716 |
| October | impact1 | Upstream | 544.694378 | 960.73619 | 8.4 | 10.2 | 10.2 | 265 | 512 | 13.0619 | 21.7393 | 0.0732 |
| October | impact1 | Downstream | 544.694378 | 960.73619 | 8 | 9.2 | 12.3 | 280 | 499 | 2.4451 | 2.1708 | 0.3872 |
| October | impact1 | Downstream | 544.694378 | 960.73619 | 8.1 | 9.1 | 11 | 280 | 497 | 21.1505 | 27.7955 | NA |
| October | impact1 | Downstream | 262.45981 | 1419.5778 | 8 | 9 | 12.1 | 281 | 500 | 21.3962 | 28.7905 | NA |
| August | impact3 | Upstream | 262.45981 | 1419.5778 | 7.85 | 9 | 15 | 214 | 429 | 12.17 | 17.04 | 0.19 |
| August | impact3 | Upstream | 262.45981 | 1419.5778 | 7.87 | 9.1 | 15 | 214 | 429 | 12.17 | 17 | 0.23 |
| August | impact3 | Upstream | 262.45981 | 1419.5778 | 8.1 | 9.2 | 14.9 | 214 | 426 | 12.59 | 17.65 | 0.23 |
| August | impact3 | Downstream | 262.45981 | 1419.5778 | 7.76 | 7.21 | 14.1 | 300 | 501 | 25.58 | 11.2 | 0.42 |
| August | impact3 | Downstream | 262.45981 | 1419.5778 | 7.76 | 7 | 14.2 | 340 | 502 | 25.5 | 11.15 | 0.48 |
| August | impact3 | Downstream | 262.45981 | 1419.5778 | 7.74 | 7.2 | 14.1 | 329 | 521 | 25.58 | 11.17 | 0.51 |
| November | impact3 | Upstream | 262.45981 | 1419.5778 | 8.4 | 10.1 | 7.4 | 250 | 414 | 11.7522 | 15.5916 | 0.017 |
| November | impact3 | Upstream | 262.45981 | 1419.5778 | 8 | 9.88 | 7 | 231 | 400 | 11.5832 | 15.8315 | 0.0296 |
| November | impact3 | Upstream | 262.45981 | 1419.5778 | 8.44 | 9.9 | 7.5 | 251 | 412 | 12.7609 | 17.1628 | 0.11 |
| November | impact3 | Downstream | 262.45981 | 1419.5778 | 7.78 | 7.99 | 10.1 | 400 | 510 | 30.8588 | 14.0238 | 0.2624 |
| November | impact3 | Downstream | 262.45981 | 1419.5778 | 7.98 | 7 | 10.2 | 399 | 501 | 32.205 | 13.942 | 0.3364 |
| November | impact3 | Downstream | 262.45981 | 1419.5778 | 7.59 | 7.7 | 10.1 | 401 | 501 | 32.0597 | 13.6228 | 0.5153 |
| October | impact3 | Upstream | 262.45981 | 1419.5778 | 8.1 | 10 | 8.7 | 192 | 383 | 11.7682 | 13.8832 | 0.062 |
| October | impact3 | Upstream | 262.45981 | 1419.5778 | 8.23 | 10.1 | 8 | 192 | 383 | 12.0071 | 13.4692 | NA |
| October | impact3 | Upstream | 262.45981 | 1419.5778 | 7.99 | 9.8 | 7.9 | 191 | 383 | 12.949 | 12.52 | NA |
| October | impact3 | Downstream | 262.45981 | 1419.5778 | 7.5 | 7.05 | 10.7 | 328 | 501 | 27.3315 | 5.4497 | 0.0496 |
| October | impact3 | Downstream | 262.45981 | 1419.5778 | 7.6 | 7.21 | 11 | 312 | 500 | 27.4088 | 5.7267 | 0.0495 |
| October | impact3 | Downstream | 262.45981 | 1419.5778 | 7.7 | 8 | 10.5 | 311 | 500 | 28.992 | 0.477 | NA |
| August | impact5 | Upstream | 616.57635 | 710.69216 | 8.14 | 7.7 | 16.5 | 278 | 557 | 12.01 | 21.7039 | 0.06 |
| August | impact5 | Upstream | 616.57635 | 710.69216 | 8.17 | 7.8 | 16.8 | 276 | 553 | 12.11 | 22 | 0.15 |
| August | impact5 | Upstream | 616.57635 | 710.69216 | 8.13 | 7.7 | 16.5 | 276 | 552 | 12.2 | 22.1571 | 0.2 |
| August | impact5 | Downstream | 616.57635 | 710.69216 | 7.12 | 7.9 | 17.5 | 290 | 580 | 21.45 | 30.1118 | 0.29 |
| August | impact5 | Downstream | 616.57635 | 710.69216 | 7 | 7.8 | 17.9 | 299 | 581 | 21.53 | 30.01 | 0.3 |
| August | impact5 | Downstream | 616.57635 | 710.69216 | 7.1 | 7.7 | 17.2 | 290 | 581 | 21.76 | 31.7572 | 0.5 |
| November | impact5 | Downstream | 616.57635 | 710.69216 | 6.9 | 7.44 | 12.4 | 596 | 580 | 20.6862 | 29.9434 | 0.0557 |
| November | impact5 | Upstream | 616.57635 | 710.69216 | 6.6 | 8.8 | 12.2 | 300 | 570 | 12.0441 | 22.7717 | 0.0434 |
| November | impact5 | Downstream | 616.57635 | 710.69216 | 7 | 7.3 | 12.5 | 595 | 600 | 21.8714 | 33.3385 | 0.4633 |
| November | impact5 | Upstream | 616.57635 | 710.69216 | 7.1 | 8.01 | 12 | 305 | 569 | 11.8387 | 22.3621 | NA |
| November | impact5 | Upstream | 616.57635 | 710.69216 | 6.8 | 8.6 | 12.3 | 301 | 560 | 12.2972 | 23.0568 | NA |
| November | impact5 | Downstream | 616.57635 | 710.69216 | 7.11 | 7.5 | 12.6 | 595 | 581 | 21.1411 | 29.8426 | NA |
| October | impact5 | Upstream | 616.57635 | 710.69216 | 7 | 9 | 12 | 285 | 570 | 12.9793 | 20.4749 | 0.0496 |
| October | impact5 | Upstream | 616.57635 | 710.69216 | 7 | 8 | 12.6 | 285 | 570 | 13.1267 | 21.1448 | NA |
| October | impact5 | Upstream | 616.57635 | 710.69216 | 7 | 8.7 | 12.3 | 285 | 575 | 13.1899 | 20.5973 | NA |
| October | impact5 | Downstream | 616.57635 | 710.69216 | 7.21 | 7.6 | 13 | 297 | 595 | 20.9988 | 27.9171 | 0.053 |
| October | impact5 | Downstream | 616.57635 | 710.69216 | 6.99 | 7.65 | 13.1 | 299 | 596 | 21.391 | 27.0066 | 0.058 |
| October | impact5 | Downstream | 616.57635 | 710.69216 | 7.1 | 7.6 | 13.6 | 299 | 599 | 21.5223 | 28.1461 | NA |
| August | impact8 | Upstream | 130.776 | 1704.7183 | 7.2 | 8.2 | 16 | 260 | 520 | 0.37 | 58.1561 | 0.37 |
| August | impact8 | Upstream | 130.776 | 1704.7183 | 7.1 | 8 | 16.7 | 251 | 510 | 0.41 | 73.01 | 0.41 |
| August | impact8 | Downstream | 130.776 | 1704.7183 | 7.6 | 6.5 | 15.5 | 354 | 679 | 27.02 | 18.3298 | 0.75 |
| August | impact8 | Downstream | 130.776 | 1704.7183 | 7.08 | 6.8 | 15 | 351 | 700 | 33.64 | 18.36 | 2.67 |
| August | impact8 | Downstream | 130.776 | 1704.7183 | 7.06 | 6.7 | 14.8 | 400 | 689 | 34.96 | 19.0705 | 3.19 |
| August | impact8 | Upstream | 130.776 | 1704.7183 | 7.21 | 8.2 | 16 | 261 | 529 | NA | NA | NA |
| November | impact8 | Downstream | 130.776 | 1704.7183 | 7 | 6.2 | 12.6 | 500 | 688 | 30.8588 | 22.2327 | 1.4165 |
| November | impact8 | Downstream | 130.776 | 1704.7183 | 7.2 | 6.21 | 12.5 | 488 | 690 | 32.205 | 22.4769 | 1.5182 |
| November | impact8 | Downstream | 130.776 | 1704.7183 | 7.05 | 6.1 | 12.4 | 488 | 698 | 32.0597 | 21.7597 | 1.5539 |
| November | impact8 | Upstream | 130.776 | 1704.7183 | 6.87 | 8.5 | 11 | 302 | 519 | 12.4457 | 0.0356 | 0.0356 |
| November | impact8 | Upstream | 130.776 | 1704.7183 | 7 | 7.9 | 11.3 | 300 | 520 | 12.7569 | 0.0555 | 0.0555 |
| November | impact8 | Upstream | 130.776 | 1704.7183 | 7 | 7.7 | 11.5 | 300 | 512 | 12.7553 | 0.1093 | 0.1093 |
| October | impact8 | Upstream | 130.776 | 1704.7183 | 7.23 | 8.6 | 11 | 233 | 512 | 31.2454 | 86.0535 | 0.3872 |
| October | impact8 | Upstream | 130.776 | 1704.7183 | 7.1 | 8 | 11.5 | 200 | 531 | 12.4005 | 16.4512 | NA |
| October | impact8 | Upstream | 130.776 | 1704.7183 | 7.12 | 8 | 11.1 | 231 | 521 | 13.7351 | 21.1614 | NA |
| October | impact8 | Downstream | 130.776 | 1704.7183 | 7.2 | 6.5 | 12.3 | 399 | 698 | 56.8351 | 61.29 | 6.5294 |
| October | impact8 | Downstream | 130.776 | 1704.7183 | 7.21 | 6.5 | 12 | 387 | 690 | 61.7349 | 71.4298 | 8.0063 |
| October | impact8 | Downstream | 130.776 | 1704.7183 | 7.1 | 6 | 13 | 370 | 688 | 80.4529 | 106.4766 | 12.5839 |

**Table S3.** Model selection, where the best model, based on (AIC) values, is highlighted in orange. * = interaction term; + = additive term; df = degrees of freedom; AIC = Akaike information criterion; BIC = Bayesian information criterion; logLIK = Log-Likelihood, **χ**^2^= Chi-squared

| **Response variable** | **Predictors** | ***df*** | **AIC** | **BIC** | **LogLIK** | **χ^2^** |
| --- | --- | --- | --- | --- | --- | --- |
| *Sewage fungus abundance* | Intercept | 3 | 1110.3 | 1117.1 | -552.15 |  |
|  | Area (i.e., presence of sewage effluent) | 4 | 1098.0 | 1107.1 | -545.00 | 14.31 |
|  | Urbanisation | 4 | 1110.1 | 1119.2 | -551.03 | 0.00 |
|  | Agriculture | 4 | 1111.3 | 1120.5 | -551.67 | 0.00 |
|  | Area+Urbanisation | 5 | 1097.9 | 1109.3 | -543.95 | 15.44 |
|  | Area*Urbanisation | 6 | 1095.1 | 1108.7 | -541.53 | 0.00 |
|  | Area+Agriculture | 5 | 1099.2 | 1110.5 | -544.59 | 0.00 |
|  | Area*Agriculture | 6 | 1097.6 | 1111.2 | -542.79 | 0.00 |
|  | Urbanisation+Agriculture | 5 | 1107.7 | 1119.0 | -548.83 | 0.00 |
|  | Urbanisation*Agriculture | 6 | 1116.7 | 1130.3 | -552.32 | 0.00 |
|  | Month | 5 | 1081.6 | 1093.0 | -535.79 | 26.09 |
|  | Area+Month | 6 | 1069.0 | 1082.7 | -528.53 | 47.59 |
|  | Area*Month | 8 | 1040.6 | 1058.8 | -512.29 | 46.37 |
|  | Urban+Month | 6 | 1081.7 | 1095.4 | -534.86 | 0.00 |
|  | Urban*Month | 8 | 1068.6 | 1068.6 | -526.30 | 0.00 |
|  | Agricultural+Month | 6 | 1083.0 | 1096.6 | -535.48 | 0.00 |
|  | Agricultural*Month | 8 | 1072.1 | 1090.3 | -528.03 | 0.00 |
| *Macroinvertebrate abundance* | Intercept | 3 | 974.85 | 981.68 | -484.42 |  |
|  | Area (i.e.,presence of sewage effluent) | 4 | 963.31 | 972.42 | -477.66 | 13.53 |
|  | Urbanisation | 4 | 978.27 | 987.38 | -485.14 | 0.00 |
|  | Agriculture | 4 | 979.63 | 988.74 | -485.82 | 0.00 |
|  | Area+Urbanisation | 5 | 966.70 | 978.08 | -478.35 | 14.93 |
|  | Area*Urbanisation | 6 | 969.64 | 983.30 | -478.82 | 0.00 |
|  | Area+Agriculture | 5 | 968.08 | 979.46 | -479.04 | 0.00 |
|  | Area*Agriculture | 6 | 972.21 | 985.87 | -480.11 | 0.00 |
|  | Urbanisation+Agriculture | 5 | 977.34 | 988.72 | -483.67 | 0.00 |
|  | Urbanisation*Agriculture | 6 | 990.21 | 1003.8 | -489.11 | 0.00 |
|  | Month | 5 | 951.26 | 962.64 | -470.63 | 26.08 |
|  | Area+Month | 6 | 939.45 | 953.11 | -463.72 | 50.76 |
|  | Area*Month | 8 | 913.35 | 931.56 | -448.68 | 46.65 |
|  | Urban+Month | 6 | 954.60 | 968.26 | -471.30 | 0.00 |
|  | Urban*Month | 8 | 959.24 | 977.45 | -471.62 | 0.00 |
|  | Agricultural+Month | 6 | 956.00 | 969.66 | -472.00 | 0.00 |
|  | Agricultural*Month | 8 | 963.24 | 981.46 | -473.62 | 0.00 |
| *Macroinvertebrate EPT Score* | Intercept | 3 | -160.67 | -153.84 | 83.337 |  |
|  | Area (i.e.,presence of sewage effluent) | 4 | -169.89 | -160.79 | 88.949 | 11.22 |
|  | Urbanisation | 4 | -146.13 | -137.02 | 77.06 | 0.00 |
|  | Agriculture | 4 | -144.73 | -135.62 | 76.36 | 0.00 |
|  | Area+Urbanisation | 5 | -154.86 | -143.48 | 82.43 | 12.14 |
|  | Area*Urbanisation | 6 | -137.336 | -123.68 | 74.67 | 0.00 |
|  | Area+Agriculture | 5 | -153.55 | -142,17 | 181.77 | 0.00 |
|  | Area*Agriculture | 6 | -134.94 | -121.28 | 73.47 | 0.00 |
|  | Urbanisation+Agriculture | 5 | -128.97 | -117.58 | 69.48 | 0.00 |
|  | Urbanisation*Agriculture | 6 | -98.49 | -84.836 | 55.248 | 0.00 |
|  | Month | 5 | -145.47 | -134.08 | 77.73 | 16.49 |
|  | Area+Month | 6 | -154.46 | -140.80 | 83.23 | 55.97 |
|  | Area*Month | 8 | -151.33 | -133.17 | 83.66 | 25.78 |
|  | Urban+Month | 6 | -130.96 | -117.30 | 71.48 | 0.00 |
|  | Urban*Month | 8 | -99.78 | -81.57 | 57.89 | 0.00 |
|  | Agricultural+Month | 6 | -129.55 | -115.89 | 70.78 | 0.00 |
|  | Agricultural*Month | 8 | -94.91 | -76.69 | 55.45 | 0.00 |
| *Macroinvertebrate Riverfly Score* | Intercept | 3 | 160.97 | 167.80 | -77.48 |  |
|  | Area (i.e.,presence of sewage effluent) | 4 | 162.30 | 171.40 | -77.14 | 0.67 |
|  | Urbanisation | 4 | 171.63 | 180.74 | -81.82 | 0.00 |
|  | Agriculture | 4 | 173.54 | 182.65 | -82.77 | 0.00 |
|  | Area+Urbanisation | 5 | 173.24 | 184.62 | -81.62 | 2.30 |
|  | Area*Urbanisation | 6 | 174.30 | 187.96 | -81.15 | 0.00 |
|  | Area+Agriculture | 5 | 175.09 | 186.47 | -82.54 | 0.00 |
|  | Area*Agriculture | 6 | 178.58 | 192.24 | -83.29 | 5.19 |
|  | Urbanisation+Agriculture | 5 | 182.55 | 193.94 | -86.28 | 0.00 |
|  | Urbanisation*Agriculture | 6 | 208.53 | 222.19 | -98.26 | 0.00 |
|  | Month | 5 | 163.06 | 174.45 | -76.53 | 19.49 |
|  | Area+Month | 6 | 164.32 | 177.98 | -76.16 | 44.21 |
|  | Area*Month | 8 | 163.39 | 181.60 | -73.69 | 15.83 |
|  | Urban+Month | 6 | 173.20 | 186.86 | -80.60 | 0.00 |
|  | Urban*Month | 8 | 199.61 | 217.82 | -91.80 | 0.00 |
|  | Agricultural+Month | 6 | 175.22 | 188.88 | -81.61 | 0.00 |
|  | Agricultural*Month | 8 | 204.59 | 229.80 | -94.29 | 0.00 |
| *Periphyton: cyanobacteria* | Intercept | 3 | 955.13 | 960.55 | -474.56 |  |
|  | Area (i.e.,presence of sewage effluent) | 4 | 937.64 | 944.87 | -464.82 | 19.49 |
|  | Urbanisation | 4 | 949.52 | 956.75 | -470.76 | 0.00 |
|  | Agriculture | 4 | 950.56 | 957.79 | -471.28 | 0.00 |
|  | Area+Urbanisation | 5 | 931.99 | 941.03 | -461.00 | 8.84 |
|  | Area*Urbanisation | 6 | 926.51 | 937.35 | -457.26 | 9.79 |
|  | Area+Agriculture | 5 | 933.02 | 942.06 | -461.51 | 0.00 |
|  | Area*Agriculture | 6 | 928.91 | 939.75 | -458.45 | 0.00 |
|  | Urbanisation+Agriculture | 5 | 941.00 | 950.03 | -465.50 | 0.00 |
|  | Urbanisation*Agriculture | 6 | 947.51 | 958.35 | -467.76 | 0.00 |
|  | Month | 4 | 938.84 | 946.06 | -465.42 | 11.73 |
|  | Area+Month | 5 | 921.42 | 930.46 | -455.71 | 19.58 |
|  | Area*Month | 6 | 900.64 | 911.48 | -444.32 | 46.87 |
|  | Urban+Month | 5 | 933.26 | 942.29 | -461.63 | 0.00 |
|  | Urban*Month | 6 | 924.77 | 935.61 | -456.39 | 0.00 |
|  | Agricultural+Month | 5 | 934.30 | 943.34 | -462.15 | 0.00 |
|  | Agricultural*Month | 6 | 927.27 | 938.11 | -457.64 | 0.00 |
| *Periphyton: green algae* | Intercept | 3 | 749.37 | 754.79 | -371.69 |  |
|  | Area (i.e.,presence of sewage effluent) | 4 | 736.61 | 743.84 | -364.31 | 14.76 |
|  | Urbanisation | 4 | 749.68 | 756.91 | -370.84 | 0.00 |
|  | Agriculture | 4 | 751.11 | 758.33 | -364.77 | 0.00 |
|  | Area+Urbanisation | 5 | 736.95 | 745.98 | -363.47 | 2.59 |
|  | Area*Urbanisation | 6 | 736.16 | 747.00 | -362.08 | 5.05 |
|  | Area+Agriculture | 5 | 738.38 | 747.41 | -364.19 | 0.00 |
|  | Area*Agriculture | 6 | 738.92 | 749.76 | -363.46 | 0.00 |
|  | Urbanisation+Agriculture | 5 | 746.32 | 755.36 | -368.16 | 0.00 |
|  | Urbanisation*Agriculture | 6 | 756.41 | 767.25 | -372.20 | 0.00 |
|  | Month | 4 | 737.54 | 744.77 | -364.77 | 13.57 |
|  | Area+Month | 5 | 724.88 | 733.91 | -357.44 | 21.45 |
|  | Area*Month | 6 | 712.06 | 722.90 | -350.03 | 44.35 |
|  | Urban+Month | 5 | 737.80 | 746.84 | -363.90 | 0.00 |
|  | Urban*Month | 6 | 735.58 | 746.42 | -361.79 | 0.00 |
|  | Agricultural+Month | 5 | 739.21 | 748.25 | -364.61 | 0.00 |
|  | Agricultural*Month | 6 | 738.37 | 749.21 | -363.19 | 0.00 |
| *Periphyton: diatoms* | Intercept | 3 | 929.28 | 934.70 | -461.64 |  |
|  | Area (i.e.,presence of sewage effluent) | 4 | 913.43 | 920.66 | -452.72 | 17.85 |
|  | Urbanisation | 4 | 925.60 | 932.83 | -458.80 | 0.00 |
|  | Agriculture | 4 | 927.00 | 934.22 | -459.50 | 0.00 |
|  | Area+Urbanisation | 5 | 909.73 | 918.77 | -449.87 | 5.44 |
|  | Area*Urbanisation | 6 | 903.97 | 914.81 | -445.98 | 8.90 |
|  | Area+Agriculture | 5 | 911.13 | 920.16 | -450.56 | 0.00 |
|  | Area*Agriculture | 6 | 907.01 | 917.85 | -447.51 | 0.00 |
|  | Urbanisation+Agriculture | 5 | 918.54 | 927.57 | -454.27 | 0.00 |
|  | Urbanisation*Agriculture | 6 | 925.08 | 935.92 | -456.54 | 0.00 |
|  | Month | 4 | 913.17 | 920.40 | -452.59 | 13.82 |
|  | Area+Month | 5 | 897.24 | 906.27 | -443.62 | 21.29 |
|  | Area*Month | 6 | 879.48 | 890.32 | -433.74 | 45.59 |
|  | Urban+Month | 5 | 909.47 | 918.51 | -449.74 | 0.00 |
|  | Urban*Month | 6 | 902.48 | 913.32 | -445.24 | 0.00 |
|  | Agricultural+Month | 5 | 910.87 | 919.90 | -450.43 | 0.00 |
|  | Agricultural*Month | 6 | 904.92 | 915.76 | -446.46 | 0.00 |
| *Nutrients:*  *nitrate* | Intercept | 3 | 608.17 | 614.96 | -301.08 |  |
|  | Area (i.e.,presence of sewage effluent) | 4 | 604.77 | 613.82 | -298.38 | 5.40 |
|  | Urbanisation | 4 | 615.62 | 624.67 | -303.81 | 0.00 |
|  | Agriculture | 4 | 616.89 | 625.94 | -304.44 | 0.00 |
|  | Area+Urbanisation | 5 | 612.25 | 623.57 | -301.13 | 6.63 |
|  | Area*Urbanisation | 6 | 620.12 | 633.69 | -304.06 | 0.00 |
|  | Area+Agriculture | 5 | 613.52 | 624.83 | -301.76 | 0.00 |
|  | Area*Agriculture | 6 | 622.72 | 636.30 | -305.36 | 0.00 |
|  | Urbanisation+Agriculture | 5 | 619.93 | 631.24 | -304.96 | 0.00 |
|  | Urbanisation*Agriculture | 6 | 636.30 | 649.88 | -312.15 | 0.00 |
|  | Month | 5 | 599.22 | 610.53 | -294.61 | 20.70 |
|  | Area+Month | 6 | 595.83 | 609.41 | -291.92 | 40.46 |
|  | Area*Month | 8 | 583.82 | 601.92 | -283.91 | 28.17 |
|  | Urban+Month | 6 | 606.73 | 620.30 | -297.36 | 0.00 |
|  | Urban*Month | 8 | 608.40 | 626.50 | -296.20 | 0.00 |
|  | Agricultural+Month | 6 | 607.99 | 621.57 | -297.99 | 0.00 |
|  | Agricultural*Month | 8 | 612.29 | 630.39 | -298.14 | 0.00 |
| *Nutrients: phosphate* | Intercept | 3 | 222.09 | 227.83 | -108.047 |  |
|  | Area (i.e.,presence of sewage effluent) | 4 | 217.71 | 225.35 | -104.85 | 6.38 |
|  | Urbanisation | 4 | 231.82 | 239.46 | -111.90 | 0.00 |
|  | Agriculture | 4 | 233.11 | 240.76 | -112.56 | 0.00 |
|  | Area+Urbanisation | 5 | 227.77 | 237.34 | -108.88 | 7.33 |
|  | Area*Urbanisation | 6 | 232.95 | 244.42 | -110.47 | 0.00 |
|  | Area+Agriculture | 5 | 229.00 | 238.56 | -109.50 | 0.00 |
|  | Area*Agriculture | 6 | 235.43 | 246.90 | -111.71 | 0.00 |
|  | Urbanisation+Agriculture | 5 | 240.56 | 250.12 | -115.27 | 0.00 |
|  | Urbanisation*Agriculture | 6 | 261.76 | 273.23 | -124.88 | 0.00 |
|  | Month | 5 | 210.58 | 220.14 | -100.14 | 29.97 |
|  | Area+Month | 6 | 204.54 | 216.02 | -96.27 | 57.21 |
|  | Area*Month | 8 | 182.40 | 197.69 | -83.19 | 43.78 |
|  | Urban+Month | 6 | 220.73 | 232.21 | -104.36 | 0.00 |
|  | Urban*Month | 8 | 228.16 | 243.46 | -106.08 | 0.00 |
|  | Agricultural+Month | 6 | 222.18 | 233.66 | -105.09 | 0.00 |
|  | Agricultural*Month | 8 | 230.75 | 246.05 | -107.37 | 0.00 |

**Table S4**. Abundance of each benthic organism analysed, divided for each area with and without sewage discharge, and by sampling month. Mean abundance is showed as µg chl-a/cm², and se represents the standard error of the mean.

| **Benthic organisms** | **Area** | **Mean abundance**  **(µg chl-a/cm²)** | ***se*** |
| --- | --- | --- | --- |
| **August** |  |  |  |
| Green Algae | *Downstream* | 44.17 | 29.78 |
|  | Upstream | 335.67 | 182.47 |
| Diatoms | *Downstream* | 5876.25 | 2245.33 |
|  | Upstream | 8947.50 | 3754.53 |
| Cyanobacteria | *Downstream* | 10633.33 | 3372.40 |
|  | Upstream | 6975.00 | 3200.00 |
| **October** |  |  |  |
| Green Algae | *Downstream* | 119.25 | 109.97 |
|  | Upstream | 595.75 | 572.40 |
| Diatoms | *Downstream* | 4755.00 | 1505.62 |
|  | Upstream | 6113.33 | 988.99 |
| Cyanobacteria | *Downstream* | 9425.00 | 3747.87 |
|  | Upstream | 10058.33 | 2452.07 |

**Figures:**


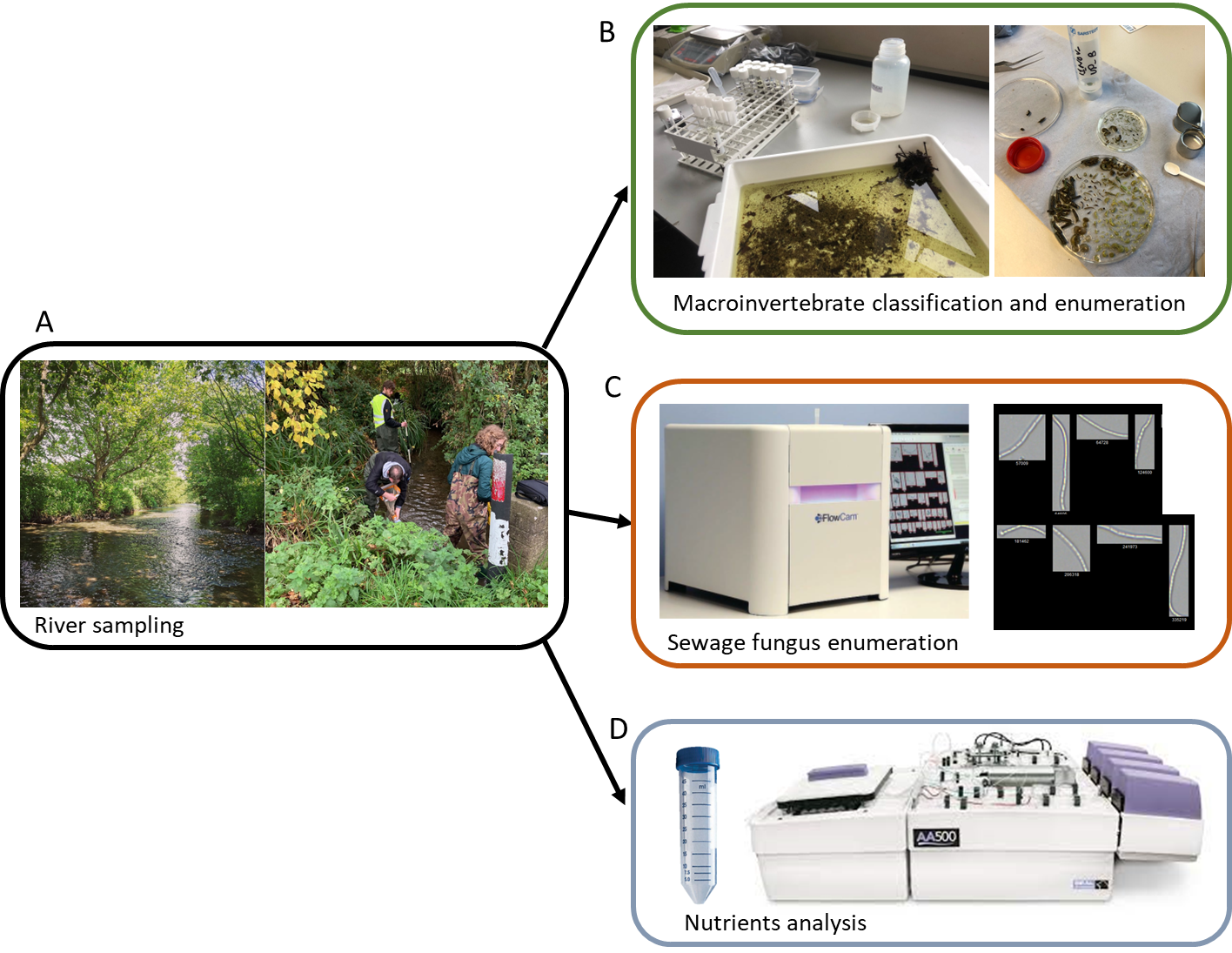


**Figure S1.** Field sampling (A) and laboratory analysis (B to D). A) River sampling collection and in-situ water parameter measurements (e.g., BenthoTorch analysis). B) macroinvertebrate classification and enumeration using the Extended Riverfly methodology and water quality scores. C) Sewage fungus enumeration using FlowCAM 8000. D) Nutrient analysis using an auto-analyser.

**Results for June month: additional sampling month for benthic organisms**


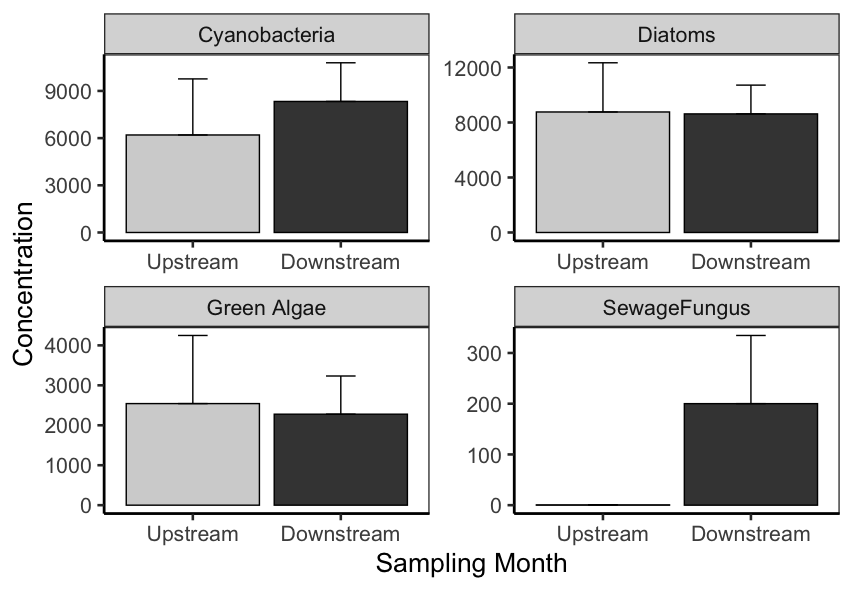


**Figure S2.** Concentration of benthic algae (Cyanobacteria, Diatoms and Green Algae) expressed in µg chl-a/cm² and concentration of sewage fungus filaments in 50mL of water sample in the month of June. Light grey represents the upstream area and dark grey the downstream area from the point of discharge of treated sewage.


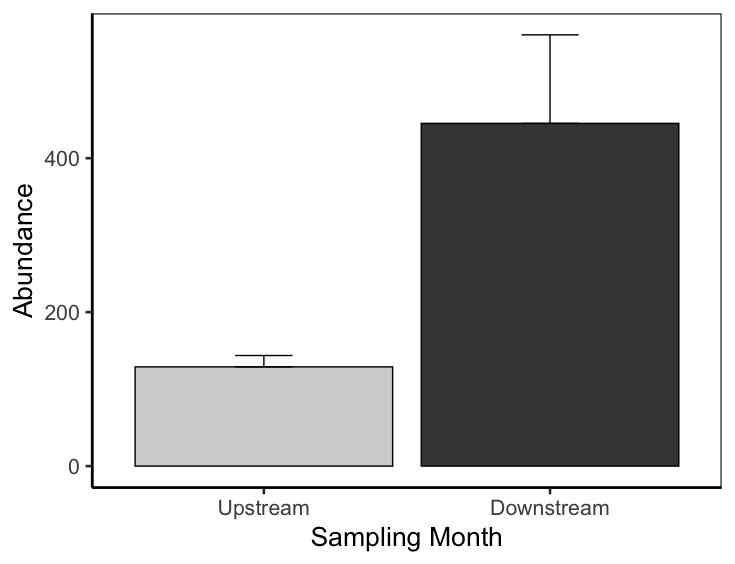


**Figure S3.** Mean abundance ± SE of macroinvertebrates, in the areas without (upstream – light grey) and with (downstream – dark grey) treated sewage discharge, in June. Abundance is expressed as individuals per 500 mL.
